# Cortex Parcellation Associated Whole White Matter Parcellation in Individual Subjects

**DOI:** 10.3389/fnhum.2017.00352

**Published:** 2017-07-06

**Authors:** Patrick Schiffler, Jan-Gerd Tenberge, Heinz Wiendl, Sven G. Meuth

**Affiliations:** Department of Neurology, University Hospital MünsterMünster, Germany

**Keywords:** white matter parcellation, diffusion tensor imaging, diffusion weighted imaging, fiber tracking, FreeSurfer, brain anatomy

## Abstract

The investigation of specific white matter areas is a growing field in neurological research and is typically achieved through the use of atlases. However, the definition of anatomically based regions remains challenging for the white matter and thus hinders region-specific analysis in individual subjects. In this article, we focus on creating a whole white matter parcellation method for individual subjects where these areas can be associated to cortex regions. This is done by combining cortex parcellation and fiber tracking data. By tracking fibers out of each cortex region and labeling the fibers according to their origin, we populate a candidate image. We then derive the white matter parcellation by classifying each white matter voxel according to the distribution of labels in the corresponding voxel from the candidate image. The parcellation of the white matter with the presented method is highly reliable and is not as dependent on registration as with white matter atlases. This method allows for the parcellation of the whole white matter into individual cortex region associated areas and, therefore, associates white matter alterations to cortex regions. In addition, we compare the results from the presented method to existing atlases. The areas generated by the presented method are not as sharply defined as the areas in most existing atlases; however, they are computed directly in the DWI space of the subject and, therefore, do not suffer from distortion caused by registration. The presented approach might be a promising tool for clinical and basic research to investigate modalities or system specific micro structural alterations of white matter areas in a quantitative manner.

## 1. Introduction

The analysis of micro structural white matter properties has become increasingly important, especially in multiple sclerosis research (Deppe et al., 2007, 2014, 2016). There are several techniques such as diffusion-weighted magnet resonance imaging (DWI) that are sensitive to white matter alterations that cannot be assessed by conventional MRI.

Korbinian Brodmann introduced the first parcellation method for the cortex in 1909 by classifying cortex areas by their cytoarchitecture (Brodmann, 1909). Through the rise of structural MRI in brain imaging, *in vivo* cortex parcellation became possible (Rademacher et al., 1992) by classifying the cortex on the basis of cortical gyri and sulci, thus providing a way to associate cortex alterations to brain functions. Automatic parcellation of the cortex was then introduced (Fischl et al., 2002, 2004; Glasser et al., 2016) and is since a heavily used tool in clinical and basic research for region specific analysis of the cortex. Further cortex parcellations were created which seek to provide a higher resolution through defining more cortex labels (Desikan et al., 2006; Destrieux et al., 2010), or to classify the cortex from functional networks derived from functional MRI (Craddock et al., 2012; Blumensath et al., 2013; Shen et al., 2013; Moreno-Dominguez et al., 2014; Thirion et al., 2014; Parisot et al., 2016).

While the parcellation of the human cortex into functionally differentiable areas can be easily performed on the basis of cortical gyri and sulci, there are no macro-anatomical landmarks that permit direct classification of the white matter. Several white matter atlases were created to overcome this problem by mapping regions directly onto images under investigation (Wakana et al., 2004, 2007; Mori et al., 2005; Hua et al., 2008; Mori et al., 2008; Oishi et al., 2008). However, the registration of these atlases relies on mapping to gray matter landmarks, as the structure of the white matter alone is insufficient.

The parcellation of white matter is usually performed by mapping a white matter atlas onto the image under investigation. These atlases are typically created by parcellating the white matter manually or semi-automatically in a group of subjects by investigating diffusion-weighted images, structural images, or fiber tracking results. For example, specialists map the fiber tracking results onto diffusion-weighted or structural images and label each white matter voxel (Mori et al., 2005). The created parcellations are then mapped into the same space where an atlas is derived from these overlaying parcellations. Generally, there are two types of atlases. Deterministic atlases like the *ICBM-DTI-81* (Mori et al., 2005; Wakana et al., 2007) assign a label to each white matter voxel that indicates the white matter area. Probabilistic atlases on the other hand, like the *JHU white-matter tractography atlas* (Hua et al., 2008) assign each white matter voxel a probability that indicates how likely a voxel belongs to a certain white matter area. These atlases are typically applied in two steps. A template, which is either a single subject or a group average, is mapped onto the image under investigation. The resulting mapping is then applied to the white matter atlas, which is in the same space as the template, to map the parcellation onto the image under investigation. However, as Bloy et al. (2012) already pointed out and Rohlfing (2013) demonstrated as an example, mapping a white matter atlas into the desired image can be error-prone since the accuracy of the white mater parcellation heavily relies on the registration to the template. Our approach aims to overcome this strong dependency on registration by parcellating the white matter directly in the space of the diffusion-weighted image. There are already approaches such as *FreeSurfer white matter parcellation* (Salat et al., 2009) that do not describe a white matter atlas, but a method that is applied in every individual subject to parcellate the white matter. However, this is a rather basic approach since it just classifies the white matter according to the nearest cortex region.

Diffusion tensor imaging (DTI) provides a base for the reconstruction of fiber tracts in the human brain. Here, we present an approach called *cortex associated individual white matter parcellation* that combines parcellation of the gray matter and fiber tracking in DTI images to permit cortex parcellation-associated whole white matter parcellation in individual subjects. The general idea of combining fiber tracking and gray matter parcellation was already outlined previously (Park et al., 2004). However, the focus of our article lies on the classification of each white matter voxel and thus the differentiation between the generated white matter areas.

## 2. Methods

This paper presents an automatic method for parcellating the whole white matter into cortex region associated areas. Therefore, cortex parcellation and deterministic fiber tracking in DTI are combined.

### 2.1. Diffusion-weighted imaging

Diffusion-weighted imaging measures the diffusion of water molecules inside the tissue in a specific direction. This is achieved through a certain parametrization of the MRI sequence. By performing multiple measurements of the diffusion in multiple directions, the general diffusion can be estimated. A detailed explanation of DWI can be found in Mori (2007).

In equally constituted tissue such as gray matter, the diffusion of water molecules is nearly isotropic. However, in the white matter, this diffusion is partially inhibited perpendicular to the fiber tracts, which leads to anisotropic diffusion. This characteristic allows for conclusions to be drawn about the orientation of the nerve fibers.

Published for the first time in 1994 (Basser et al., 1994), DTI relies on a mathematical model that describes the measured diffusion in every voxel and has been established as a common standard in neurological research. The model regards diffusion as a second order tensor that can be visualized as an ellipsoid. For undirected diffusion, this tensor consists of six parameters that can be derived from the DWI images. The linearized diffusion tensor is described by three orthogonal eigenvectors that determine the ellipsoids location and circumference. The longest of these vectors is usually called the main diffusion direction. If the diffusion tensor mainly describes isotropic diffusion, it takes the shape of a sphere, whereas for anisotropic diffusion, the tensor can take the shape of a cigar or a coin. A detailed explanation of DTI can be found in Mori (2007).

In DTI images of the human brain, spherically-shaped tensors are mainly located in the gray matter or the cerebrospinal fluid (CSF), whereas cigar-shaped tensors occur mainly in the white matter.

### 2.2. Cortex parcellation

There are several different strategies for parcellations of the cortex. Two of the parcellations commonly used in the FreeSurfer software package (Dale et al., 1999; Fischl et al., 1999, 2004) are the Desikan-Killiany Atlas (Desikan et al., 2006) and the Destrieux Atlas (Destrieux et al., 2010). Both atlases are developed for automated cortex labeling based on the gyri of the cortex and they are both anatomically valid and reliable (Desikan et al., 2006; Destrieux et al., 2010). Desikan et al. published the Desikan-Killiany Atlas in 2006 and Destrieux et al. published the Destrieux Atlas in 2010. Through employing the Desikan-Killiany Atlas, the cortex of each hemisphere is parcellated into 34 regions of interest. The Destrieux Atlas provides a finer granulated parcellation as it parcellates each hemisphere into 74 regions of interest. To use these parcellations, the recon-all script from the FreeSurfer software collection (Dale et al., 1999; Fischl et al., 1999, 2004) is employed on the structural (T1) MRI image. Its output includes the parcellation with the Desikan-Killiany Atlas, with the Destrieux Atlas, as well as a segmentation of the subcortical areas (nuclei). This procedure as well as the pre-processing of the structural images was already performed in the provided datasets. Both atlases were chosen for testing the presented method because they are widely used as part of FreeSurfer and were already included in the provided datasets. Furthermore, in the provided datasets, both the Desikan-Killiany Atlas as well as the Destrieux Atlas were already mapped into the space of the diffusion-weighted images.

### 2.3. Fiber tractography

One field of application for DWI is the reconstruction of nerve fibers in the human brain. These fibers are located in the white matter and cannot be assessed through structural MRI images. It can be assumed that the cigar shape of the tensor inside the white matter is caused by the inhibition of water molecule diffusion by the myelinated axons (Assaf and Pasternak, 2008). As a result, the fibers in the human brain can be reconstructed by following the main diffusion direction of the tensors (Conturo et al., 1999).

To overcome noise and artifacts in the DTI images, several more complex fiber tracking approaches were developed (see Feigl et al., 2014 for review) like the class of probabilistic fiber tracking algorithms (Parker et al., 2002; Behrens et al., 2003, 2007). These algorithms choose the propagation direction for the fibers with a probability derived from the underlying diffusion model, which makes them robust against noise. Through their probabilistic nature, the results of these fiber tracking algorithms are not exactly reproducible and therefore can include a degree of uncertainty into the test of the presented method. Hence, for the purpose of white matter parcellation, we use the Fiber Assignment Continuous Tracking (FACT) method (Mori et al., 1999) that is included in the Diffusion Toolkit (Wang et al., 2007) and is established as a common standard for deterministic fiber tracking. FACT was employed on the datasets with the default parametrization of the Diffusion Toolkit, which is an automatic mask threshold and an angle threshold of 35° as stopping criteria.

### 2.4. White matter parcellation

**Figure 1:**
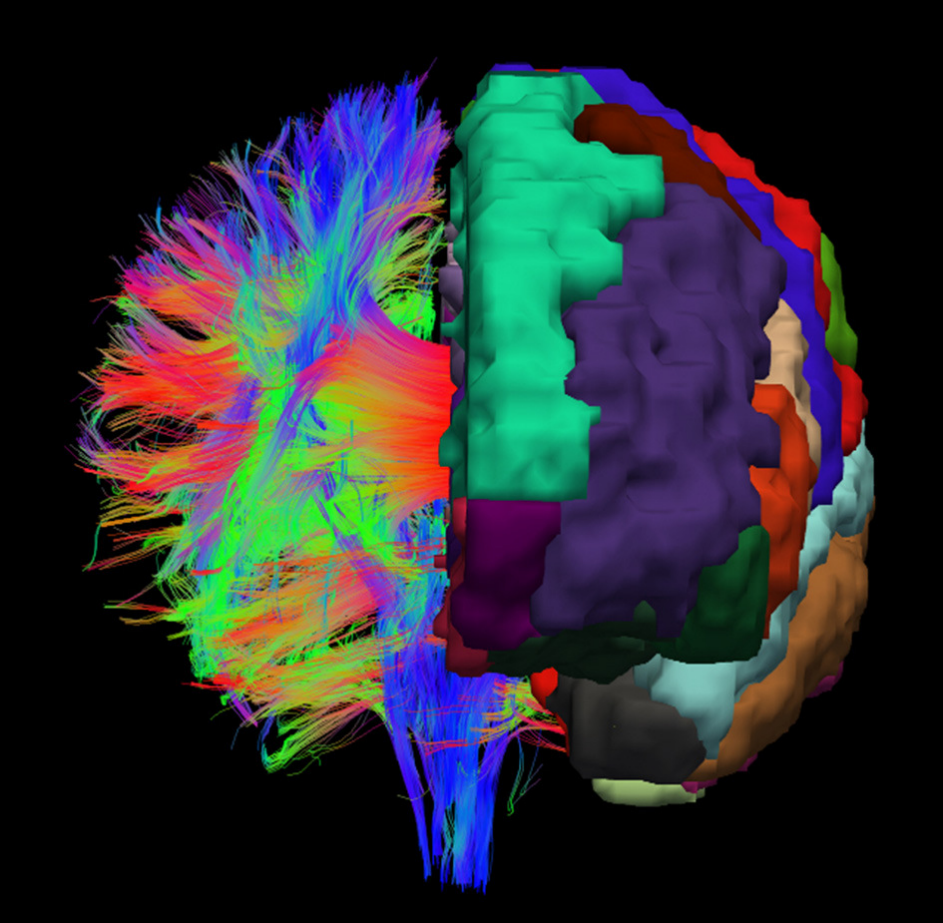
Example visualization of the cortex parcellation mapped onto the fiber tracking results. Only the main fibers are shown. These data are the input for the actual white matter parcellation.

For achieving a parcellation of the whole white matter into cortex region associated areas, cortex parcellation and fiber tracking are combined. An example for the registered cortex parcellation into the tract space is shown in Figure 1. Due to the fact that the fiber tracts are mainly symmetrical, it is possible to imagine how the reconstructed fibers connect the different gray matter regions, especially the cortex areas.

For the actual white matter parcellation, the fibers are tracked out of every cortex region and labeled. Each part of the fiber that lies in the white matter is labeled with the same label that the cortex parcellation assigned to the start voxel. After the fiber labeling is done, a list is generated that contains the label count for every voxel of the white matter. In the next step, every fiber is tracked a second time. While a fiber is tracked, the label it received in the previous step is written into a list that is associated to the voxel where the fiber section is present. A single voxel usually contains numerous fibers and, therefore, these lists contain a count for every possible cortex label that can be written into this voxels associated list. A probability is then assigned to every label a list contains. The label with the highest probability then determines the chosen label for a specific voxel.

In detail, let L be the list of labels and $L_{i}$ the quantity of the label at position *i* of the list. Since the probability is computed for every cortex label, all lists have the same length |L| and additionally a label has the same position in every list. The local probability $p_{i}^{L}$ for a specific label is then

(1) $$p_{i}^{L}=\frac{L_{i}}{\sum_{j=0}^{\left|L\right|}L_{j}}$$

In addition to this local label probability, the label probabilities of the neighboring voxels are also taken into account for determining which label is assigned to the specific voxel. To cover this, 1 is extended as follows. Let *N* = {(*x y z*)^*T*^}\(0 0 0)^*T*^ with −1 ≤ *x, y, z* ≤ 1 the set of the relative positions to the neighboring voxels and therefore $L^{N}$ the label list for these voxels. The probabilities of the neighboring voxels for a given list element at index *i* are weightily taken into account with:

(2) $$p_{i}^{N}=\sum_{n\in N}\|n{\|}_{2}^{-1}\frac{L_{i}^{n}}{\sum_{j=0}^{\left|L\right|}L_{j}^{n}}$$

The overall probability for a label to get assigned to a specific voxel is therefore:

(3) $$p_{i}=w^{L}*p_{i}^{L}+w^{N}*p_{i}^{N}$$

with 0 ≤ *w*^*L*^, *w*^*N*^ ≤ 1 and *w*^*L*^ + *w*^*N*^ = 1.

To explain this equation, the local probability for a specific label to be chosen is the number of occurrences of the label divided by the aggregated number of label occurrences in the current voxel. For the neighboring voxels that are also considered for the label assignment, the probability is computed in the same way. However, these probabilities are not evenly taken into account since the neighboring voxels have different distances to the local voxel. Therefore, the neighboring voxels are weighted through their distance to the local voxel. Finally the two parts, the local probability and the aggregated probability of the neighboring voxels, are weighted with two parameters (*w*^*L*^ and *w*^*N*^) to adjust the influence of the two parts.

Therefore, the expansion of *p*_*i*_ yields the computation in every voxel as:

(4) $$p_{i}=w^{L}*\frac{L_{i}}{\sum_{j=0}^{\left|L\right|}L_{j}}+w^{N}*\sum_{n\in N}\frac{L_{i}^{n}}{\|n{\|}_{2}\sum_{j=0}^{\left|L\right|}L_{j}^{n}}$$

**Figure 2:**
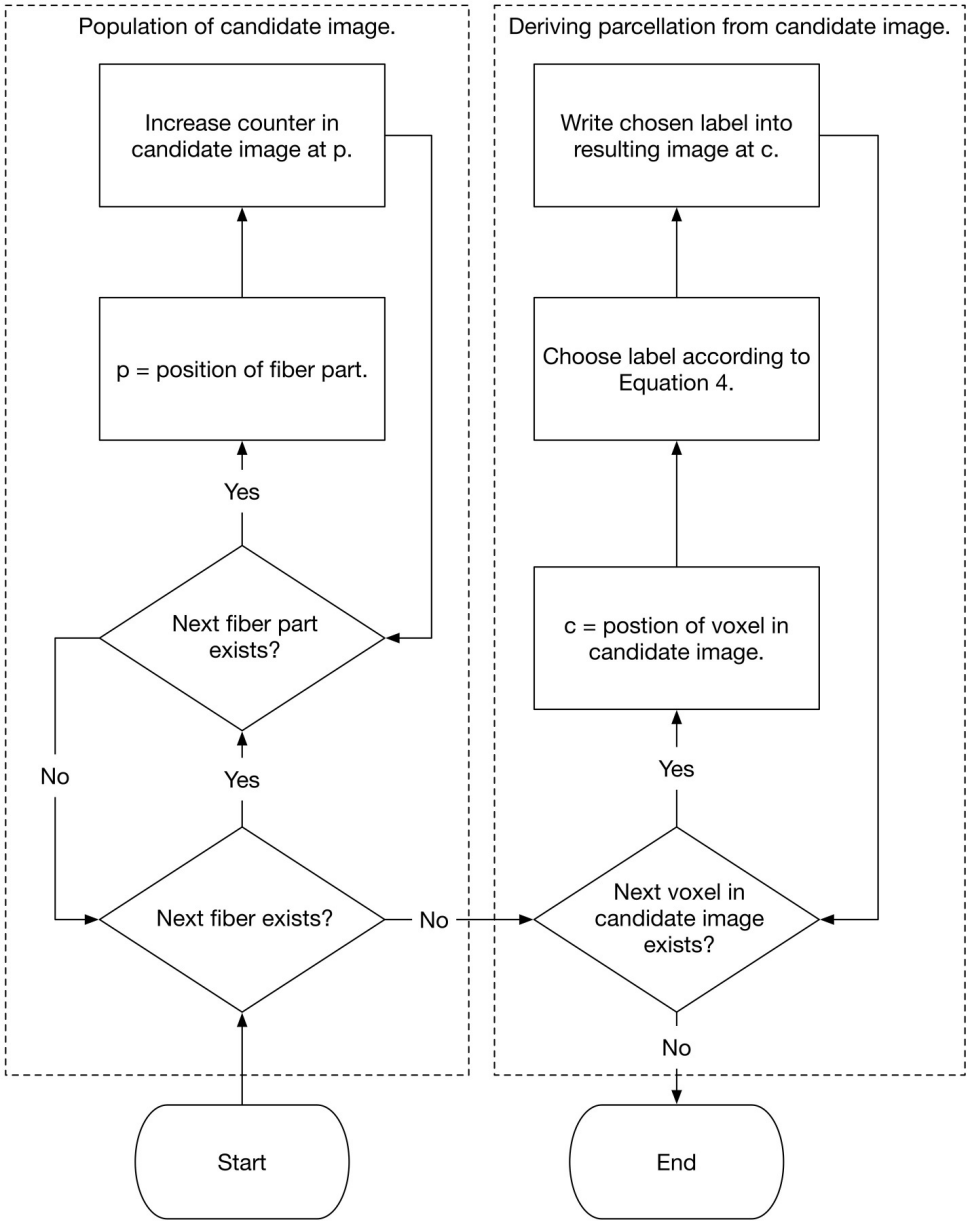
Flow chart that shows the population of the candidate image as well as the selection of the white matter labels.

The label with the highest probability in a specific voxel is then assigned to this voxel. By performing this procedure for every white matter voxel, the cortex associated individual white matter parcellation is generated. Figure 2 demonstrates the method schematically as a flow chart. The actual implementation of the algorithm is written in Rust (www.rust-lang.org) (Schiffler et al., 2016) and is freely available for download on our GitHub page (neuro.github.io).

### 2.5. Data

We employed the method on 78 datasets from the freely available WU-Minn Human Connectome Project (HCP) collective (Van Essen et al., 2013). The HCP data release includes high-resolution 3T MR scans from young healthy adult twins and non-twin siblings (ages 22–35) as structural images (T1w and T2w) (Milchenko and Marcus, 2013) and high angular resolution diffusion images (dMRI) (Sotiropoulos et al., 2013). The diffusion data were already preprocessed with the HCP diffusion pipeline (Jenkinson et al., 2002, 2012; Andersson et al., 2003; Fischl, 2012; Glasser et al., 2013; Andersson and Sotiropoulos, 2015, 2016) (updated with EDDY 5.0.10). The datasets further include structural preprocessed data with the HCP structural pipeline (Jenkinson et al., 2002, 2012; Fischl, 2012; Glasser et al., 2013), including FreeSurfer and PostFreeSurfer pipeline outputs.

All used diffusion data have a voxel size of 1.25 *mm* × 1.25 *mm* × 1.25 *mm* and a FOV of 210 *mm*. Diffusion weighting consisted of 3 shells of $b=1000\frac{s}{mm^{2}}$, $b=2000\frac{s}{mm^{2}}$, and $b=3000\frac{s}{mm^{2}}$ with approximately 90 diffusion directions plus 6 *b* = 0 images on each shell. Additionally, an inverted phase encoding direction for each shell was acquired.

## 3. Results

**Figure 3:**
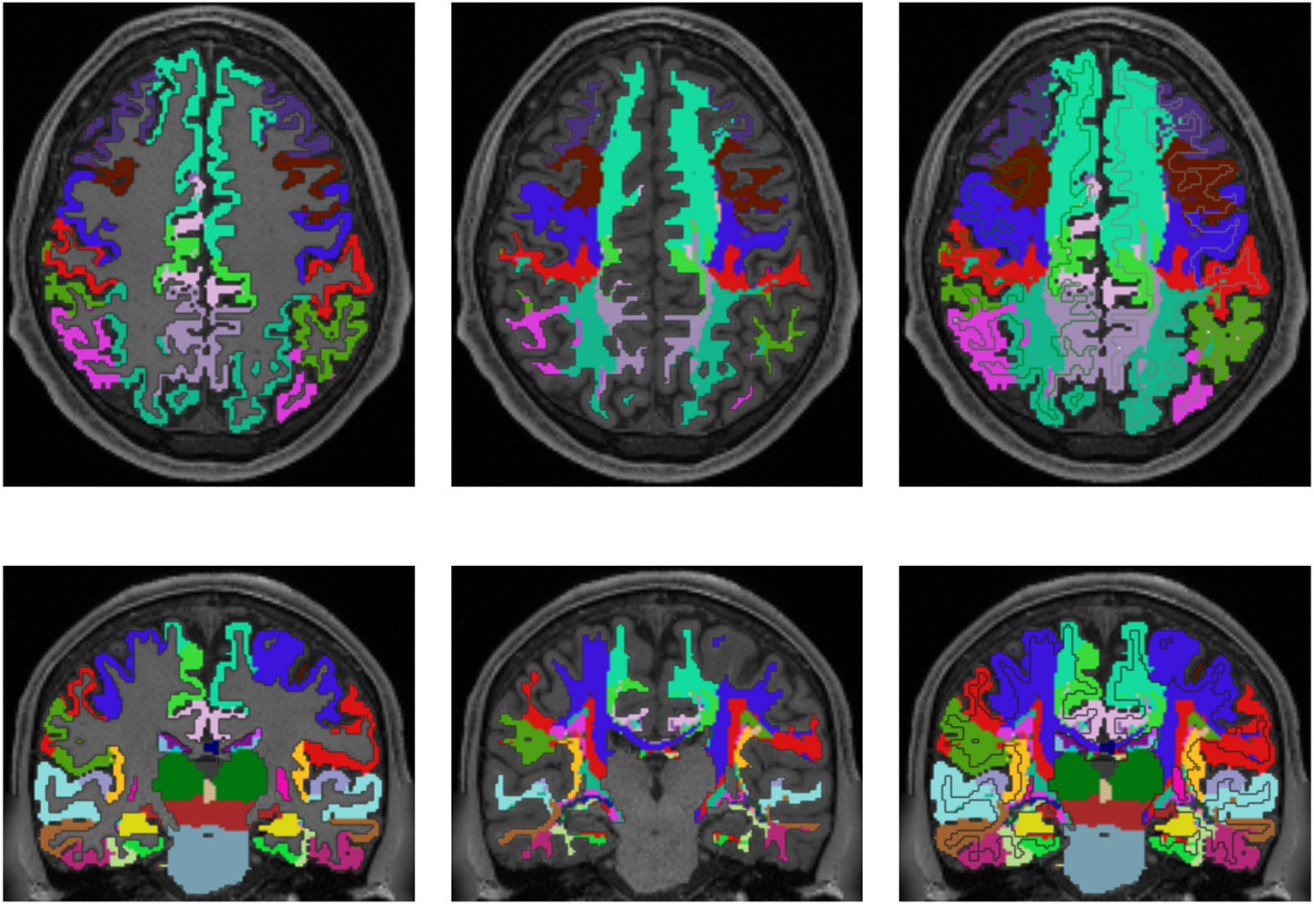
Comparison of cortex parcellation with the Desikan-Killiany Atlas, gray matter segmentation, and the resulting individual white matter parcellation shown in an axial and a coronal slice of a single subject. Column one: Cortex parcellation and gray matter segmentation mapped into the structural image which was used to generate the parcellation and segmentation. Column two: Resulting white matter parcellation of the developed method mapped into the same structural image as in column one. Column three: Images of column one and two mapped into one image. The black lines indicate the boundaries between the cortex areas and the white matter areas.

**Figure 4:**
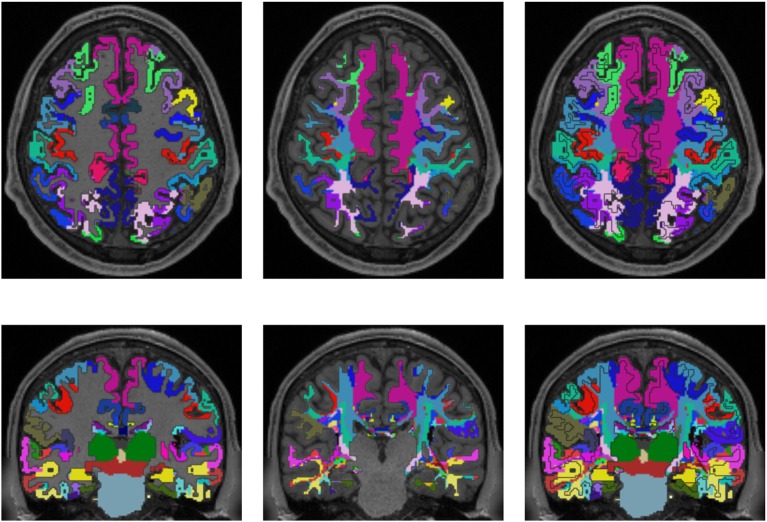
Comparison of cortex parcellation with the Destrieux Atlas, gray matter segmentation, and the resulting individual white matter parcellation shown in an axial and a coronal slice of a single subject. Column one: Cortex parcellation and gray matter segmentation mapped into the structural image which was used to generate the parcellation and segmentation. Column two: Resulting white matter parcellation of the developed method mapped into the same structural image as in column one. Column three: Images of column one and two mapped into one image. The black lines indicate the boundaries between the cortex areas and the white matter areas.

Figures 3, 4 show the results of the cortex parcellation, the gray matter segmentation, as well as the results for the developed individual white matter parcellation method. An axial slice and a coronal slice are shown in both figures. The images in the first column show the resulting cortex parcellation as well as the gray matter segmentation overlaid over the structural MRI image; the white matter is faded out. The images in the second column show the actual result of the developed white matter parcellation method. Notice that the images in the second column look similar to the images in column one, but here with colored regions contained within the white matter. These regions are distinguished through the same colors as the cortex regions and are, furthermore, associated to the cortex regions through these colors. However, the resulting white matter regions are partially not as sharply defined as the cortex regions. The third column of Figures 3, 4 shows how the resulting white matter regions are associated to the cortex regions. Column three shows the overlay of the structural image, the cortex parcellation, the gray matter segmentation, and the resulting individual white matter parcellation. It can be seen that a white matter area next to a specific cortex area has the same color as the cortex area. This means the white matter area is associated to this cortex area.

**Figure 5:**
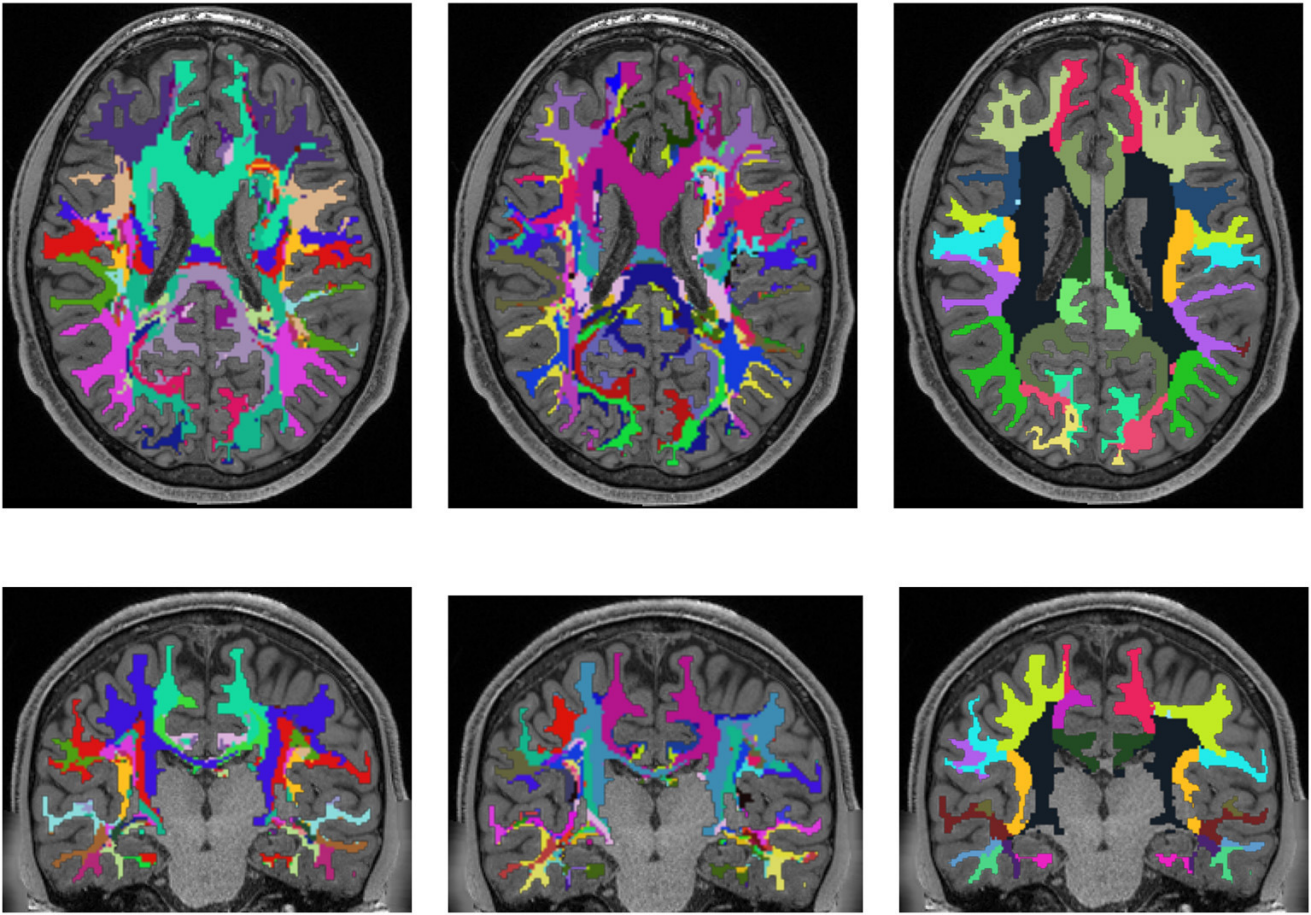
Comparison of the presented white matter parcellation method with the Desikan-Killiany Atlas for cortex parcellation, the Destrieux Atlas for cortex parcellation, and the white matter parcellation of FreeSurfer in a single subject. All parcellations are mapped into the structural image which was used to generate the parcellation. Column one: Parcellation with Desikan-Killiany Atlas for cortex parcellation. Column two: Parcellation with Destrieux Atlas for cortex parcellation. Column three: White matter parcellation of FreeSurfer.

Figure 5 shows a comparison of the results of the white matter parcellation with both cortex parcellations to the white matter parcellation produced by FreeSurfer. The FreeSurfer white matter parcellation is already included in the provided data and classifies each white matter voxel according to its nearest cortex area. Therefore, it is not a white matter atlas, but like our presented method, it describes a white matter parcellation that is computed in every individual subject. Since FreeSurfer's white matter parcellation uses the Desikan-Killiany Atlas for determining the white matter labels, it does look similar to the parcellation from our presented method, which also uses the Desikan-Killiany Atlas. However, the developed method does not classify the white matter based on the distance to cortex regions, but on the originating cortex region of the fiber tracking results.

**Figure 6:**
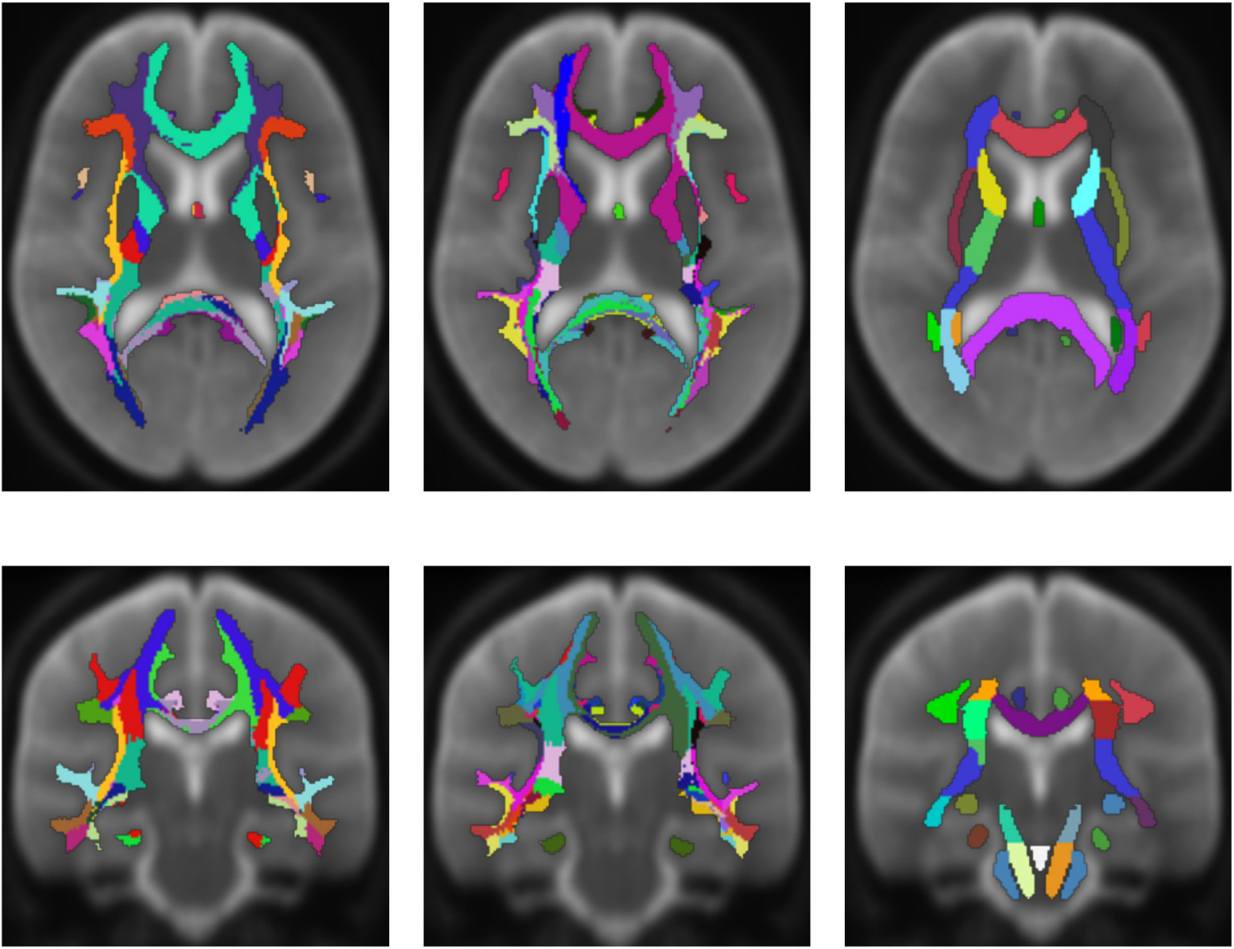
Average white matter parcellation derived from all 78 subjects with the Desikan-Killiany Atlas for cortex parcellation and the Destrieux Atlas for cortex parcellation compared to the ICBM-DTI-81 white matter atlas. Column one: Average parcellation with Desikan-Killiany Atlas for cortex parcellation. Column two: Average parcellation with Destrieux Atlas for cortex parcellation. Column three: ICBM-DTI-81 white matter atlas.

Figure 6 shows an averaged white matter parcellation with both cortex parcellations compared to the ICBM-DTI-81 white matter atlas. Therefore, all 78 subjects were mapped into the space of the atlas. The averaging was done over all 78 subjects through majority voting which simply counts for every voxel the appearance of every label and choses the label with most appearances. The ICBM-DTI-81 atlas was produced by hand segmentation of a standard-space average of diffusion MRI tensor maps from 81 subjects and contains 48 white matter tract labels. The comparison shows that the parcellations of the presented method and the ICBM-DTI-81 atlas divide the white matter in partially congruent areas. The parcellation using the Desikan-Killiany Atlas for cortex parcellation looks similar to the ICBM-DTI-81 atlas although the white matter regions the presented method produces are not as sharply defined as the regions in the ICBM-DTI-81 atlas. The parcellation with the Destrieux Atlas contains more different regions (74) in comparison to the ICBM-DTI-81 atlas and therefore offers a finer granulated parcellation. The ICBM-DTI-81 atlas only parcellates the main white matter tract, whereas the presented method generates a whole white matter parcellation.

**Figure 7:**
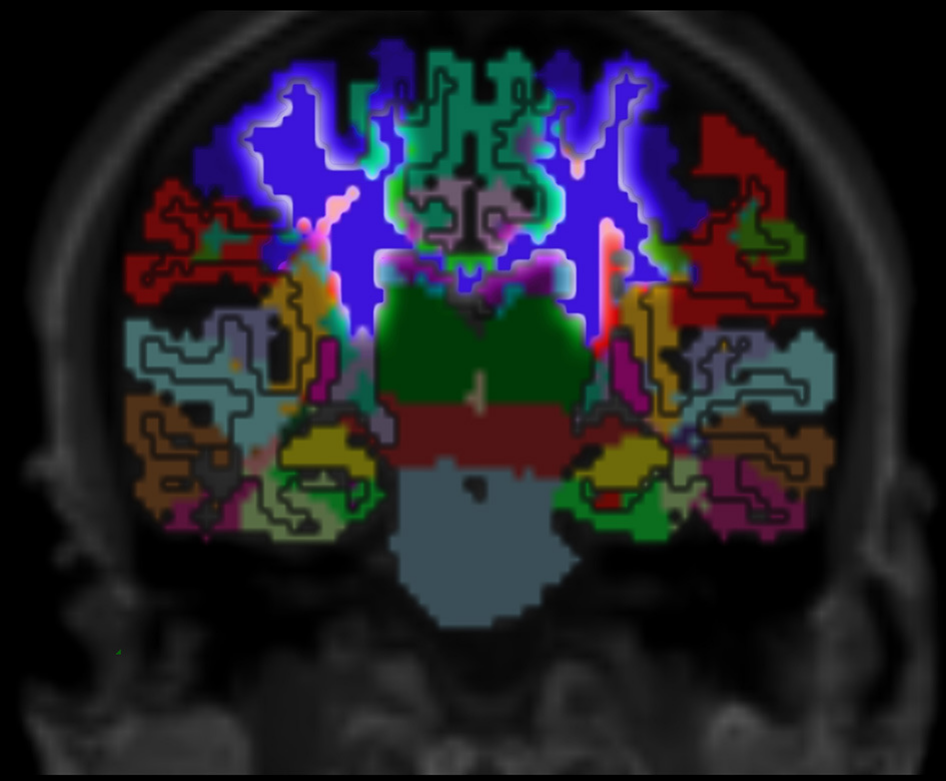
Cortex parcellation, gray matter segmentation and resulting white matter parcellation mapped into one image. The region that outlines the pre-central cortex associated white matter is highlighted. This region connects the pre-central cortex (dark blue) and the thalamus (dark green) which is known as an actual fiber pathway (Sommer, 2003; Drenckhahn, 2004).

## 4. Discussion

The developed method can be used to parcellate the whole white matter into individual cortex region associated areas. This allows for the association of white matter alterations to the originating cortex regions.

The method tends to label voxels close to the cortex with the same label as the closest cortex region. Since the fibers protrude out of the cortex, it is clear that a large number pass through the voxels that are closer to the cortical region of origin. Additionally, the greater the distance from the cortex, the more unsharp the resulting parcellation becomes. This is due to the fact that more distant areas are typically crisscrossed by a lot of fibers with different origins, and that all of these fibers have to be taken into account for choosing the resulting label. Hence, it could be useful to adapt the presented method in the future to generate a probabilistic parcellation.

Compared to the FreeSurfer white matter parcellation, the results of the presented method look similar in areas that are close to the cortex. This is because FreeSurfer's parcellation classifies each white matter voxel based on the closest cortex region. Furthermore, the FreeSurfer parcellation uses the Desikan-Killiany Atlas for determining the white matter labels that we, among other atlases, also included for generating the white matter parcellation. However, in deeper white matter areas, the presented method shows a much higher resolution of parcellation compared to the FreeSurfer white matter parcellation. This is because it labels the white matter according to the origins of the fiber tracts that are not necessarily the closest cortex regions, especially in the deeper white matter.

The presented method can be applied even in diffusion-weighted images with larger voxel size. However, an increase in voxel size can lead to an increased number of fibers with different origins per voxel and, therefore, to a higher number of candidate labels in a single voxel. A high number of candidate labels in a voxel in turn can bring uncertainty to the label voting since the method choses the label with the highest probability, even if there are multiple candidate labels with a probability close to the highest.

Since diffusion tensor imaging is a rather basic approach to model diffusion, especially in areas with crossing fibers, it could be useful to implement other diffusion models like HARDI (high angular resolution diffusion-weighted imaging) or higher-order tractography models like CSD (constrained spherical deconvolution) into the method that can model crossing fibers more accurately than DTI. As the presented method relies heavily on fiber tracking, which in turn relies on the underlying diffusion model, it is expected that using better diffusion models can lead to a more accurate white matter parcellation.

Validation of the presented method remains difficult, but one approach could be a MRI scan of an *ex vivo* brain followed by a histological analysis of the white matter fiber tracts to compare the results of the presented method to those obtained by histological investigation. However, the presented results show several properties that match with published data. For example, it is known that specific fiber pathways connect the pre-central gyrus (blue in Figure 3) with the thalamus (dark green in Figure 3) (Sommer, 2003; Drenckhahn, 2004). Using the presented method, we show in Figure 3 that these fiber pathways lead to a specific region (blue in Figure 3). This finding is also highlighted in Figure 4. Thus, the generated white matter parcellation permits a region specific analysis in structural or diffusion-weighted MRI within the white matter.

The presented method shows significant less dependency on registration than white matter atlases. Since the method uses registration just for defining seed regions in the cortex and parcellates the white matter directly in the DWI space, the resulting white matter regions do not suffer from distortion through registration.

Recent studies on patients with multiple sclerosis demonstrated thalamic atrophy even in the earliest stage of the disease (Krämer et al., 2015; Deppe et al., 2016). However, until now it remained unclear to which degree the thalamic volume loss is associated with modality specific white matter alterations. The presented approach might be a promising tool for clinical and basic research to investigate modalities or system specific micro structural alterations of white matter areas in a quantitative manner.

## Author contributions

PS: Developer of the method and main author of the manuscript. JT, HW, and SM: Provided feedback and advisory duties.

### Conflict of interest statement

The authors declare that the research was conducted in the absence of any commercial or financial relationships that could be construed as a potential conflict of interest.
